# Designing UAV Swarm Experiments: A Simulator Selection and Experiment Design Process

**DOI:** 10.3390/s23177359

**Published:** 2023-08-23

**Authors:** Abhishek Phadke, F. Antonio Medrano, Chandra N. Sekharan, Tianxing Chu

**Affiliations:** 1 Conrad Blucher Institute for Surveying and Science, Texas A&M University-Corpus Christi, Corpus Christi, TX 78412, USA; 2 Department of Computer Science, Texas A&M University-Corpus Christi, Corpus Christi, TX 78412, USA

**Keywords:** UAV, swarm, robotics, simulation

## Abstract

The rapid advancement and increasing number of applications of Unmanned Aerial Vehicle (UAV) swarm systems have garnered significant attention in recent years. These systems offer a multitude of uses and demonstrate great potential in diverse fields, ranging from surveillance and reconnaissance to search and rescue operations. However, the deployment of UAV swarms in dynamic environments necessitates the development of robust experimental designs to ensure their reliability and effectiveness. This study describes the crucial requirement for comprehensive experimental design of UAV swarm systems before their deployment in real-world scenarios. To achieve this, we begin with a concise review of existing simulation platforms, assessing their suitability for various specific needs. Through this evaluation, we identify the most appropriate tools to facilitate one’s research objectives. Subsequently, we present an experimental design process tailored for validating the resilience and performance of UAV swarm systems for accomplishing the desired objectives. Furthermore, we explore strategies to simulate various scenarios and challenges that the swarm may encounter in dynamic environments, ensuring comprehensive testing and analysis. Complex multimodal experiments may require system designs that may not be completely satisfied by a single simulation platform; thus, interoperability between simulation platforms is also examined. Overall, this paper serves as a comprehensive guide for designing swarm experiments, enabling the advancement and optimization of UAV swarm systems through validation in simulated controlled environments.

## 1. Introduction

UAV (Unmanned Aerial Vehicle) swarms refer to a collective group of UAVs that operate in a coordinated manner to achieve a common goal. UAV swarms are characterized by their ability to exhibit coordinated behaviors, where individual UAVs collaborate and interact with one another to accomplish tasks efficiently and effectively. This enables UAV swarms to perform complex operations for applications in surveillance [1,2], agriculture [3], military [4], search and rescue missions [5], and environmental monitoring [6,7]. 

The use of swarms offers several advantages over single UAVs, including increased robustness, redundancy, scalability, and enhanced mission capabilities. By leveraging swarm intelligence and advanced coordination algorithms, UAV swarms have the potential to revolutionize various industries and applications, opening up new possibilities for autonomous and collaborative aerial operations. Current swarm development can be broadly classified by their topology [8,9], by type of swarm agents such as homogeneous [10] or heterogeneous [11], and by application-specific usage such as remote sensing [12]. Of course, more advanced swarm descriptions and classifications exist that are created with a specific development goal in mind.

The environment that these swarms work in is prone to disruptions, which can impede their operation. With the degree of close-knit topology built into these swarms, damage or failure of swarm agents can result in a compromise in mission progress [13]. Additionally, swarms have a multilayered architecture of various components working cohesively to bring about acceptable operation. The creation of resilient UAV swarms requires the integration of multiple components [14]. Researchers across the globe are creating novel methods for engineering resiliency in UAV swarms [15]. For safety and reliability, it is necessary to perform rigorous testing of swarm systems in all phases of development. A wide range of general robotic or UAV specific simulation platforms exists that may be explored for this. Choosing a suitable simulator is important, as different simulation environments offer different performance, model detail, and built-in features, all of which may affect the success and the merit of a simulation-based study. Despite numerous options to choose from, there is a lack of descriptive research that categorizes and indexes them for the convenience of future experiment designers. 

There are a multitude of tools available in the robotics development scenario, such that it is almost impossible to keep track of all of them without studies like this one to track and describe use-case scenarios. Each simulator tool and the platforms they are built on have their strengths and weaknesses, which the developer must be aware of before choosing a simulator. However recent trends in development have shifted focus in terms of the compatibility of these tools, such that a lot of these tools can be used in conjunction with each other. It is now entirely possible to connect integral as well as third-party plugins from different platforms to talk to each other for data sharing and system design. This leads to advantages such as distributing simulation tasks among various platforms. While this may not necessarily increase simulation performance, it opens up a wider array of capabilities and features during system design and control. The domain of mobile robot simulation, design, and development is a growing field along with its associated subcategories. The authors in [16] portray swarm robotics as a subclass of mobile robots.

The objectives of this study are to present a descriptive case study of UAV swarm simulation tools that the authors have used to design experiments and validate results. However, not all simulation platforms were capable enough to support the expectations of all experiments. Hence, a variety of tools were needed to perform them. Sometimes interfacing the various tools for real-time and post-processed data exchange was also necessary. Additionally, this study highlights the experimental design process required to conduct both real-world and simulation tests of several UAV swarm experiments. While making progress on specific objectives in UAV swarm development that require the design of a variety of experiments, the authors have examined and used a range of simulators capable of expressing UAV swarms. The experiment designs were aimed at incorporating resiliency into UAV swarms. 

The three objectives for resilient UAV swarm development currently being worked on as part of building resilient UAV swarms by the authors are as follows:

(1) Creating inter-agent and global policies for path planning, swarm movement, and collision avoidance using techniques such as artificial potential fields and bioinspired pheromone maps.
(2) Creating swarm agent-specific SAR (Search and Rescue) frameworks that focus on improving operational swarm resilience rather than external operations.
(3) Examining the impacts to swarm dynamics and performance on the introduction of heterogeneous agents in the UAV swarm.

This study describes a concise experiment design strategy executed while creating resilient swarm protocols for various UAV swarm scenarios. These experiments were planned and categorized in an experimental series each having unique objectives and outcomes. The major contributions of this research paper include a thorough examination of existing simulation platforms, aiding researchers in selecting the most suitable tool for their swarm experimentation needs. Additionally, the outlined experimental design process will provide a valuable framework to assist users in selecting the appropriate simulation tool for developing specific objectives for UAV swarms. Presently, surveys concerning simulation platforms offer general overviews of these platforms without delving into specific case scenarios. The designed experiment series herein are implemented for the choice of simulation platforms, and sample experimental results are shown.

The novelty of this research lies in its systematic approach to designing experiments for UAV swarm systems. While the development of UAV swarm technology has gained traction due to its diverse applications, there has been a lack of standardized experimental procedures to validate and optimize the performance of these systems. This research bridges the gap by offering a well-defined and rigorous experimental design process, encompassing the selection of appropriate simulation platforms and the formulation of specific objectives. By addressing the need for robust experimental validation before deploying UAV swarms in dynamic environments, this study contributes to the advancement and resilience of swarm technology, paving the way for its successful integration in real-world applications. Researchers and practitioners can utilize the insights from this paper to conduct evidence-based evaluations, ensuring the reliability and efficiency of UAV swarm systems across various scenarios and challenges.

Our findings are condensed in this brief article below and organized in the following manner. Section 2 outlines previous work on simulation platforms and lists reasons to re-examine them. Section 3 briefly defines swarm characteristics such as network topology, deployment strategies, and composition. Section 4 describes the experimental series and their targeted development objectives. Section 5 shows implementation scenarios of the selected simulation platforms for every experiment series. Section 6 outlines future work considerations and Section 7 has a concluding statement.

## 2. Background and Motivation

A structured framework design for important UAV swarm processes, such as experiment design and validation, is of paramount importance for several reasons. Firstly, it provides a systematic and organized approach to tackle complex problems and research questions. By following a well-defined framework, researchers can establish clear objectives, identify variables, and establish a coherent plan, ensuring that their experiments yield reliable and meaningful results. Secondly, a structured framework enhances the reproducibility and comparability of experiments. With a detailed and standardized methodology, other researchers can replicate the study to validate its findings or build upon the existing knowledge, fostering scientific progress and collaboration. Furthermore, a well-designed framework helps in minimizing bias and errors in the experimental process [17]. By carefully considering potential confounding factors and controlling variables, researchers can enhance the accuracy and validity of their conclusions, strengthening the overall credibility of their work. Lastly, a structured approach aids in identifying limitations and potential pitfalls early in the research process. By incorporating rigorous validation procedures and statistical analyses, researchers can gain a deeper understanding of the robustness of their results, acknowledge any shortcomings, and suggest areas for further investigation. Thus, a structured framework design experiment design and validation is a cornerstone of reliable and impactful research. It fosters clarity, reproducibility, objectivity, and efficiency, ultimately advancing knowledge and facilitating evidence-based decision making in various domains.

A search for recent articles focused on UAV simulator review yielded only a few results and are outlined in Table 1. A comparison of three simulation tools for UAV use is described in [18]. While this is a more concise approach, the authors in [19] present a broader review of the various platforms available. Article [20] further expands the review process by considering 17 different simulation platforms to review. Article [16] presents a broad view of swarm robotics, simulators, hardware, and behavior.

During the course of the literature review, it was observed that multiple tools mentioned in past literature are now deprecated and, thus, were not included in Table 1. As technology rapidly evolves, previous studies may not reflect the current state-of-the-art in terms of available simulators and their capabilities. Tools such as USARSim [22] have pre-2020 development dates and are no longer updated. Additionally, tools such as VREP [23] have changed names, whereas Microsoft AirSim [24] while still having workable versions has been archived in favor of future projects. Additionally, as observed in [25], many researchers create custom scenario-based simulation packages for testing certain components. These lack code reusability and further support. Even currently acceptable broad-range simulation platforms may not satisfy all requirements for the objective study [26].

Therefore, there is a pressing need to create an updated and comprehensive list of UAV simulators that encompasses the latest advancements in the field. This list will serve as a valuable resource for researchers, developers, and practitioners, providing them with accurate and relevant information to choose the most suitable simulator for their specific needs. Researchers can stay informed about the latest options and make informed decisions when it comes to simulation-based studies and the development of UAV systems.

**Figure 1:**
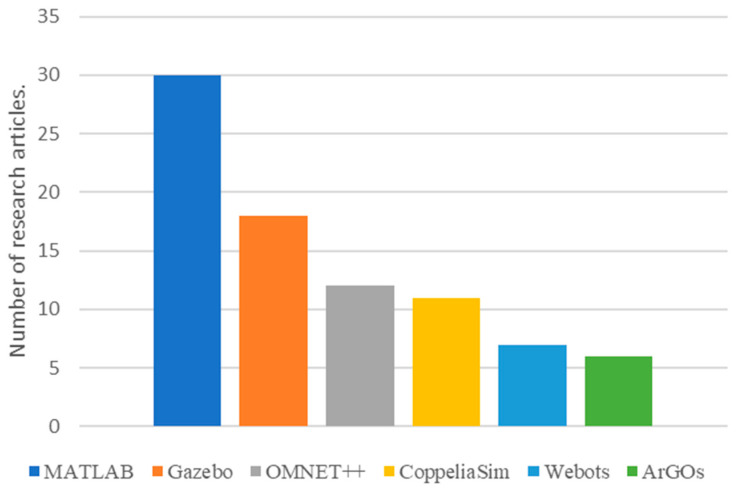
The number of studies examined using different simulation platforms over the past five years (2019–2023).

Article [27] in its literature review survey concludes that MATLAB was the most widely used simulation platform for UAV swarm experiments. However, their methodology compares items such as Java against Visual Studio, which are a programming language and an IDE, respectively. Our search parameters were more concentrated and considered simulation platforms only. Based on our analysis, we have summarized our findings in the following figures and tables. Figure 1 shows the top six platforms that were used by researchers working on aerial robotic swarms in the past five years. Table 2 presents additional information about simulation platforms examined such as OS support and cross-platform availability. Table 3 lists notable publications on swarm robotic development in the past three years (2021–2023) along with the simulation platform used for experiments and result validation.

## 3. Generalized Swarm Process Consideration during Development

It is vital to understand swarm characteristics before experiment design. Based on the three research objectives highlighted in the introduction, the experiment series will be created to observe and validate the performance of developed resilient mechanisms for the major swarm processes as discussed below.

### 3.1. Network Topologies and Communication

Network topologies and communication play a crucial role in the coordination and effectiveness of UAV swarm systems. The selection and design of appropriate network topologies significantly impact the swarm’s ability to exchange information, make collective decisions, and execute coordinated actions. Various topologies, such as centralized, decentralized, and hybrid architectures [57], can be employed based on the specific requirements and constraints of the swarm application. 

**Figure 2:**
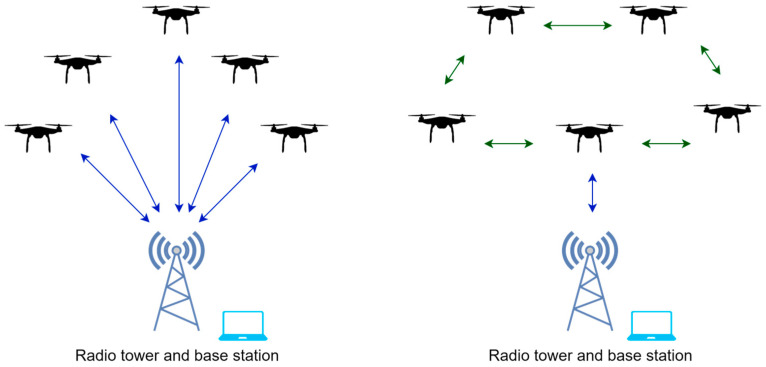
Centralized topology (**left**) and decentralized topology (**right**) for a swarm of UAV agents.

Centralized topologies, with a central controller, facilitate efficient communication and decision making but may suffer from single points of failure. Decentralized topologies distribute the decision-making across multiple UAVs, promoting resilience [58] and scalability but introducing challenges in synchronization and coordination. Figure 2 shows a centralized communication topology on the left and a decentralized communication topology on the right. 

Hybrid topologies combine centralized and decentralized elements to strike a balance between efficiency and robustness. Communication protocols and mechanisms, such as direct or indirect communication, broadcast, or multi-hop routing, determine how UAVs exchange information and collaborate within the swarm. Efficient communication protocols must address issues such as packet loss, latency, and bandwidth constraints to ensure reliable and timely data transmission. Additionally, incorporating advanced techniques like adaptive routing [59], dynamic network reconfiguration, and cognitive radio systems [60] can further enhance the swarm’s communication capabilities. Future research should focus on developing efficient network topologies and communication protocols tailored to the specific needs of UAV swarm systems, considering factors such as scalability, robustness, energy efficiency [61], and adaptability to dynamic environments. By improving network topologies and communication, UAV swarms can achieve enhanced coordination, cooperation, and performance, enabling them to tackle a wide range of complex tasks and operate effectively in diverse scenarios.

### 3.2. Deployment Strategies

Deployment strategies [62] play a critical role in the effective utilization of UAV swarms over an ROI (Region of Interest). The choice of strategy depends on various factors, including the mission objectives, environmental conditions, available resources, and desired outcomes [63]. One commonly employed strategy is the grid-based deployment approach, where the ROI is divided into a grid pattern and UAVs are strategically positioned at predefined grid points. While the decomposed grid may be any polygon [64], four-sided cells are the most common. A collection of such cells is called a subgrid [65]. Depending on a use case scenario these subgrids may be of different shapes and dimensions. Grid-based decomposition ensures comprehensive coverage of the entire area and facilitates systematic exploration of the ROI. Deploying agents to assigned grids or subgrids is particularly useful in scenarios that require even distribution of surveillance or data collection efforts, such as monitoring large agricultural fields or conducting wide-scale search and rescue operations.

**Figure 3:**
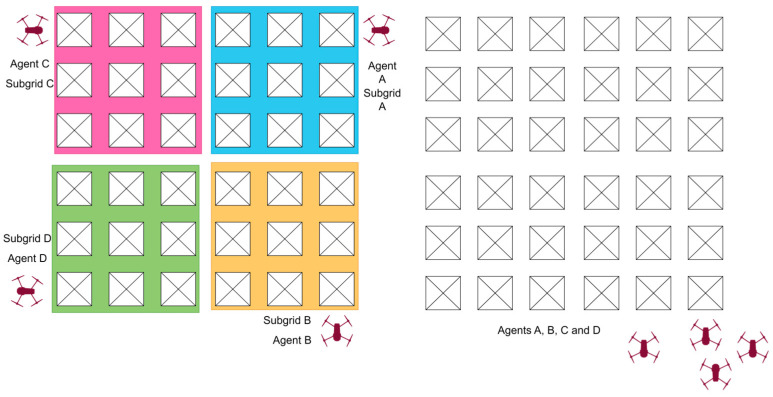
Predefined agent deployment (**left**); single point deployment (**right**).

Another deployment strategy is targeted deployment, which involves placing UAVs at specific locations within the ROI based on the mission requirements. This strategy allows for a more focused approach, concentrating the swarm’s resources on critical areas of interest. For instance, in disaster management scenarios, UAVs can be deployed near disaster-stricken regions or potential danger zones to gather detailed information, assess damage, or provide real-time situational awareness to aid response teams in their decision-making process. Figure 3 shows examples of deployment strategies that were visualized during the experiment design process. Predefined agent deployment assumes the initial placement of agents at selected points in the ROI; randomized deployment uses a single take-off point and is then relined on a randomized search by agents backed by consensus for target detection. On the left, the different colors indicate that each agent was preassigned its subgrid to search. On the right, randomized deployment shows all agents flying from a single take-off point, with no pre-assignment of subgrids.

**Figure 4:**
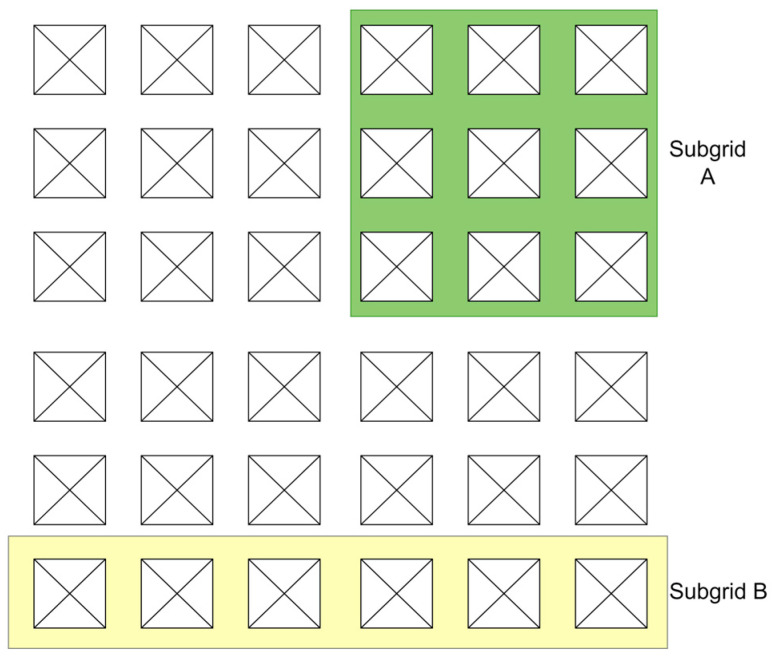
Possible subgrid selections are shown as A or B.

Figure 4 shows the two possible subgrid selections highlighted in yellow and green. These are just examples. In practice, the size and shape can be defined by the operator depending on factors such as agent capability, sensor range, coverage, or ROI geographical features. These subgrids can be selected initially and stay constant or may change dynamically across the mission timeline.

Randomized deployment strategies introduce variability and unpredictability into the swarm’s positions. By using algorithms that generate random or pseudo-random deployment patterns, the swarm can explore the ROI in a non-deterministic manner. This strategy can be beneficial in scenarios where it is essential to reduce predictability, enhance resilience, or counteract potential adversarial efforts to anticipate the swarm’s behavior. Randomized deployment strategies can be particularly useful in surveillance and security applications, where diverse and unpredictable movement patterns can make it more challenging for adversaries to identify and track the swarm’s activities.

In certain dynamic scenarios, adaptive deployment strategies come into play. These strategies involve dynamically adjusting the swarm’s configuration based on real-time information and changing mission requirements. By leveraging feedback from sensors, communication networks, or external data sources, the swarm can adapt its deployment pattern, alter its flight paths, or redistribute resources [66] within the ROI. Adaptive deployment strategies enhance the swarm’s flexibility, responsiveness, and ability to prioritize and allocate resources based on evolving situational demands. This adaptability is especially valuable in scenarios with dynamic events, such as monitoring traffic congestion, tracking fast-moving targets, or responding to emerging threats in real-time.

The choice of deployment strategy depends upon careful consideration of mission objectives, environmental factors, available resources, and the desired level of coverage and redundancy. By selecting an appropriate deployment strategy, UAV swarms can maximize their effectiveness, optimize data collection or surveillance efforts, and accomplish the intended mission goals within the ROI.

### 3.3. Swarm Formation Control Strategies

Swarm formation control strategies are fundamental to achieving coordinated behavior and desired spatial arrangements within UAV swarms. These strategies involve designing algorithms and mechanisms that enable the swarm agents to self-organize and maintain specific formations while dynamically adapting to changes in the environment or mission requirements. Various approaches, such as potential fields, artificial potential functions, behavior-based methods, distributed consensus algorithms, and network-to-distance strategies can be employed to govern swarm formation control. 

Potential field-based methods utilize attraction and repulsion forces to guide agents toward desired positions while avoiding collisions. Artificial potential functions [67] define a mathematical representation of the desired formation and drive the swarm toward it. Behavior-based methods focus on defining individual agent behaviors and interactions that collectively result in the desired formation. Distributed consensus algorithms [32,68] enable agents to reach a consensus on their positions and orientations through local interactions and information exchange. These strategies typically rely on local sensing, communication, and decision-making capabilities to achieve the desired formations without relying on centralized control. 

A network-to-distance formation controller in a UAV swarm operates on the principle that agents in close proximity should have a stronger network link between them. This relationship allows the network strength to serve as a representation of the distance constraints between two agents. By setting a network strength threshold, the controller can establish and maintain the desired formation among the UAVs. The controller continuously monitors the network connectivity and measures the strength of communication links between neighboring agents. When the network strength between two agents exceeds the predefined threshold, it indicates that the agents are within the desired distance range. In this case, the controller maintains their current positions to preserve the formation. However, if the network strength falls below the threshold it signifies that the agents are too far apart, potentially violating the desired formation. The controller then initiates appropriate control actions, such as adjusting the agents’ velocities or orientations, to bring them back within the desired distance range. By leveraging the network strength as a surrogate for distance, the network-to-distance formation controller enables coordinated movement and formation control in a UAV swarm. It allows the swarm to dynamically adapt to environmental changes or mission requirements. This controller facilitates robust and scalable coordination among the UAVs, enabling them to collectively perform complex tasks and accomplish objectives efficiently and effectively.

The choice of formation control strategy depends on factors such as the desired formation shape, scalability, robustness, and computational complexity. By enhancing swarm formation control strategies, UAV swarms can achieve precise spatial arrangements, coordinated movements, and cooperative behaviors, enabling them to effectively tackle a wide range of tasks, including surveillance, mapping, and collaborative sensing in diverse real-world scenarios.

### 3.4. Swarm Composition and Vehicle Characteristics

Heterogeneous UAV swarms, composed of UAVs with different capabilities, have gained significant attention in the field of swarm robotics due to their potential to enhance swarm performance in various applications [69]. By combining UAVs with diverse functionalities, such as different sensor payloads, communication capabilities, or task-specific capabilities, heterogeneous swarms offer increased versatility and adaptability compared to homogeneous swarms [70]. The inclusion of heterogeneous agents is predicted to surpass the performance set by homogeneous agent swarms as well as pave the way for coevolutionary abilities [71].

One significant advantage of heterogeneous UAV swarms is their ability to efficiently accomplish complex tasks through task specialization. By assigning specific roles or functions to different types of UAVs within the swarm, the workload can be distributed effectively, leading to improved task completion times. For instance, UAVs equipped with high-resolution cameras can focus on detailed surveillance and target identification, while UAVs with heavy-lift capabilities can handle payload transport or deployment tasks. This division of labor enhances the overall efficiency and enables the swarm to tackle tasks that would be challenging for a homogeneous swarm. A UGV (Unmanned Ground Vehicle) and UAV combination can work together to produce survey maps from different perspectives [72] or cooperative surveillance [73].

Moreover, heterogeneous UAV swarms demonstrate improved coverage capabilities [74]. The diverse range of sensors and payloads in heterogeneous swarms enables them to gather more comprehensive and diverse data about the environment. This enhanced coverage facilitates better situational awareness, enabling the swarm to make informed decisions and respond effectively to dynamic environmental changes. Additionally, the heterogeneous nature of the swarm allows for optimized resource allocation, as UAVs with specific capabilities can be strategically deployed in areas where their expertise is most valuable. This targeted deployment ensures efficient resource utilization and maximizes the coverage area, ultimately improving the overall effectiveness of the swarm.

**Figure 5:**
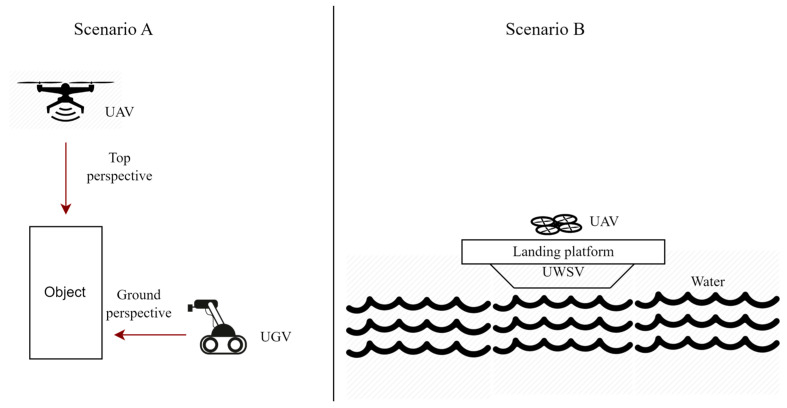
Two scenarios showing cooperation operation between heterogeneous robots.

Another critical aspect influenced by heterogeneous UAV swarms is fault tolerance. The inclusion of different types of UAVs with redundant or complementary capabilities enhances the swarm’s resilience in the face of individual UAV failures. In the event of a malfunction or system failure, other UAVs within the heterogeneous swarm can compensate for the loss by taking over the failed UAV’s responsibilities. This fault-tolerant behavior increases the reliability and robustness of the swarm, ensuring the continuity of mission execution even in challenging or unpredictable scenarios. Figure 5 shows two possible scenarios where cooperative behavior between heterogeneous agents is enabled. Scenario A shows applications where UAV and UGV work together to produce multi-perspective maps of environments. Scenario B shows a UWSV (Unmanned Water Surface Vehicle) equipped with a landing platform [75]. Such deployments have been popularly used in marine search and rescue scenarios where the landing platform allows UAVs to recharge or synchronize data between flights, thus extending their capability and flight time.

However, the integration of heterogeneous UAVs also presents challenges that must be carefully addressed. Communication and coordination between different UAV types become crucial factors in maintaining the coherence and efficiency of the swarm. Effective communication protocols and coordination mechanisms must be developed to enable seamless collaboration and exchange of information between heterogeneous UAVs. Furthermore, the heterogeneity of the swarm necessitates sophisticated task allocation and decision-making algorithms to optimize resource allocation and task assignments based on individual UAV capabilities and mission objectives. The incorporation of UAVs with diverse capabilities enhances the swarm’s efficiency, adaptability, and resilience. However, addressing communication, coordination, and task allocation challenges become imperative to fully harness the potential of heterogeneous UAV swarms. 

A UAV-specific simulation platform may not be the best choice for the design and validation of heterogeneous swarms, particularly if the participation of swarm agents over a variety of operational spaces is needed. This highlights the need for platforms with cross-platform interfacing as well as generalized tools that are capable of simulating a wide range of robots. A systematic experimental design process should be tailored specifically for validating the resilience and performance of UAV swarm systems exhibiting the above swarm process and application-specific usage. 

## 4. Simulator Selection and Experimental Design

A set of experiments were formulated to consider the assessment of the aforementioned simulation platforms and the specific research objectives. While complete experiment descriptions are not included in this article, a series categorization was created for each one. The selection of the most appropriate simulation platform was made for each experiment series to ensure an optimal environment for conducting the experiments. The experiment series and their characteristics along with the selected platform are outlined in Table 4.

Each experiment series addresses specific aspects of UAV behavior, communication, and performance. ES1 focuses on observing fast swarm phenomena, specifically flocking maneuvers and 2D path planning. The goal is to study the collective behavior of swarms and their ability to navigate efficiently in two-dimensional space. Experiments observing swarm phenomena such as flocking maneuvers and 2D path planning were initiated here. MATLAB was the choice of simulation tool used due to its robust environment for algorithm development and simulation, with specialized toolboxes for robotics and control systems. Developers also have the option to transfer created deployments into in-house higher-level modules such as Simulink and UAV toolbox. Cross-platform abilities allow interfacing with community-created packages such as the RYZE TELLO application to control Tello EDU swarms or other simulations such as CoppeliaSim. ES2 was used for observing FANET (Flying Ad Hoc Networks) and MANET (Mobile Ad Hoc Networks) topology and performance for developing inter-agent communication and routing protocols [76,77]. The choice of simulation platform was OMNET++ and associated plugins. 

ES1 and ES2 focus on critical swarm developments and on producing fast accurate results using bare simulation mechanisms. Use of high-level graphical components was limited because scenario design for swarms in a pure MATLAB based implementation is overly complex. Additional support from toolboxes, external simulator platforms, and graphic engines would be required. These methods were instead used starting from ES3. Additionally, the introduction of realistic environments such as 3D UAVs and environment models may increase the computational load, shifting the focus away from crucial observations by creating unnecessary bottlenecks. ES3 involved designing and observing photorealistic single UAVs in associated environments. Different cameras and sensors were added to the UAV, and their impact on the overall performance and capabilities of the UAV was examined through simulations. The choice of platform was the UAV toolbox in MATLAB accompanied by Simulink and the MATLAB inspired RflySim platform.

ES4 focused on examining basic movement and specific operations of the UAV swarm. Inter-swarm communication policies and swarm topology developed in ES2 were used to understand coordination and cooperation among UAVs in a swarm. The choice of platforms used was Webots and CoppeliaSim. Webots, much like CoppeliaSim has excellent support for the creation of UAV swarms and associated topologies. While the newer versions of CoppeliaSim have a generic UAV frame with modifiable physical characteristics and the ability to mount sensors such as vision and ultrasonic sensors, GPS, and LiDAR, Webots has two specific UAV models. They are Crazieflie [78] and the DJI Mavic 2 Pro [79]. Both allow the use of MATLAB scripts to function as agent-specific or global swarm controllers. ES5 implemented ground terrain features and realistic static and dynamic obstacles.

**Figure 6:**
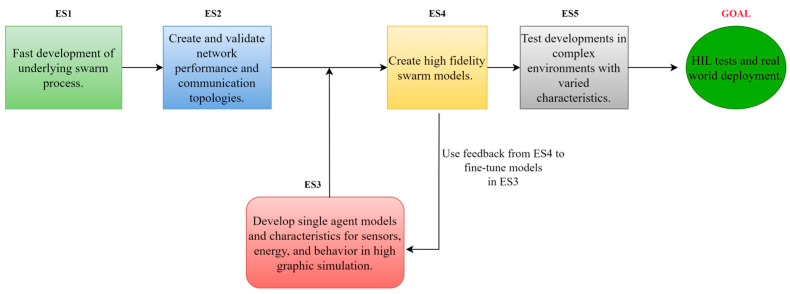
A suggested workflow using the above-proposed experiment series to design a comprehensive robust swarm.

In this series, ground terrain features and realistic obstacles were implemented in the simulations. The focus was on defining buildings, trees, and heterogeneous agents to create a realistic environment for UAVs. This allowed for the study of UAV navigation and obstacle avoidance strategies. CoppeliaSim has so far proven to be the best choice for this series with the ability to define random and specific behavior for obstacles, including physics, as well as porting control and network parameters from previous ES. Figure 6 shows the swarm development process flow using the above-mentioned experiment series.

## 5. Experiment Descriptions

**Figure 7:**
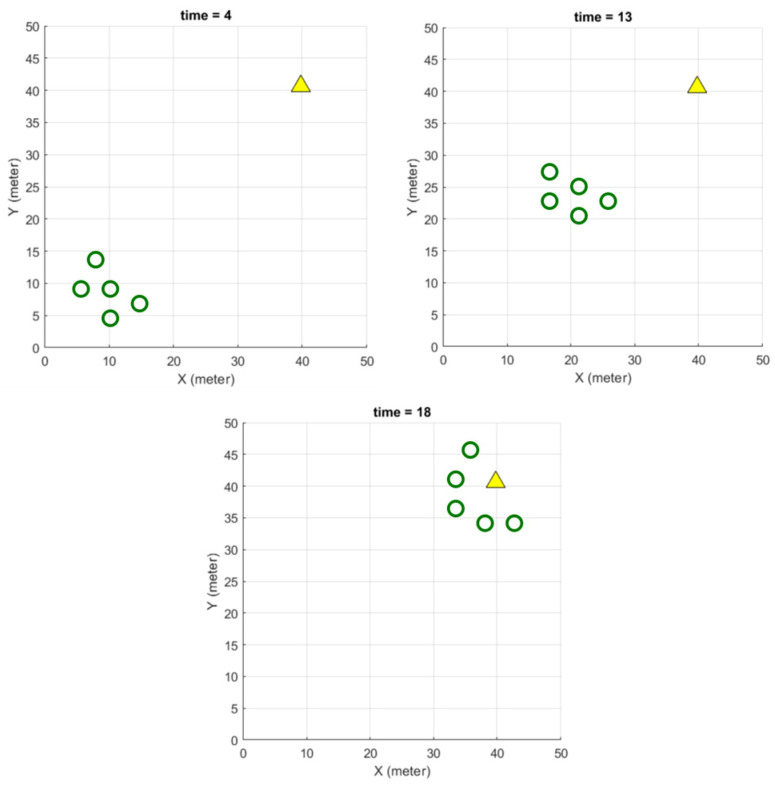
Using MATLAB to model five simple agents navigating with two programmed characteristics: formation control using network strength and target convergence using basic pheromone strategy moving on a fixed target in a 2D map. Triangles are targets, circles are agents.

This section shows the preliminary experiment progress in the various simulation platforms mentioned above. Quick 2D swarm simulations in MATLAB such as agent consensus and formation control in application scenarios such as target convergence using distance-to-network formation controller and pheromone techniques [80]. Figure 7 shows a swarm of five agents moving to a target and carrying out an encircling process. While basic results are possible using pure MATLAB, supported applications such as the UAV Scenario Designer extend functionality.

**Figure 8:**
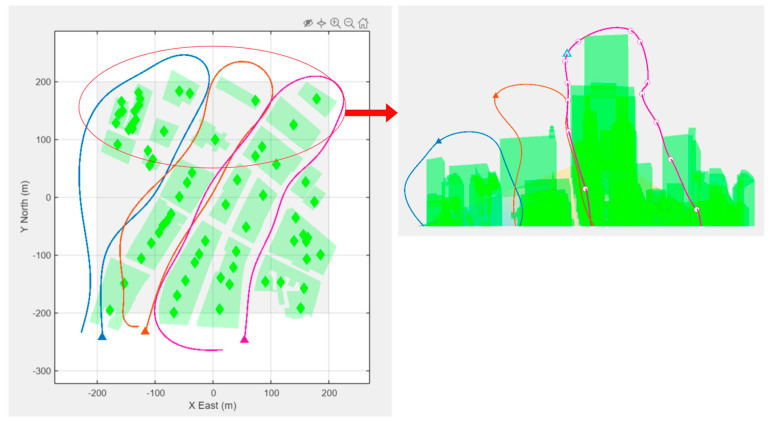
A MATLAB simulation of a top-down view of three agents and selected trajectory for a city block survey, and a sectional 3D view of the agents at their respective highest trajectory section.

Figure 8 shows a swarm of three homogeneous fixed-wing agents that were programmed to survey a city block. The experiment examined the relationship between trajectory point selection and flying altitude on agent fuel efficiency. Using minimum overhead graphics to examine essential swarm constraints is made possible using the ES1 process.

**Figure 9:**
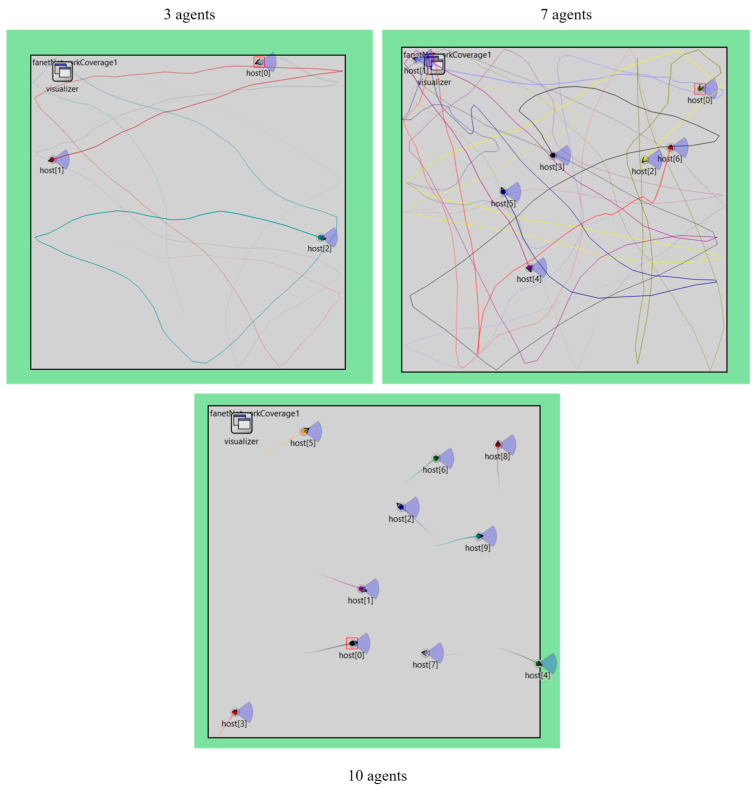
A swarm of three, seven, and ten UAV agents for area coverage using OMNET++.

The UAV swarm development domain is large enough that multiple components are needed and requires separate development and testing. It is easier to test standalone components in isolated scenarios before implementation with other components. ES2 involves the creation and testing of the underlying swarm network. FANET topologies may be based on previous MANET deployments. However, the former has been specifically designed to undergo more severe dynamic changes in node movement than a MANET. Additionally, MANET may be constricted to movement in two dimensions, with the rate of altitude change being very slow. OMNET++ allows the modeling of mobile nodes implemented with UAV characteristics and network underlay. Simulation of network node phenomenon for clustering, multi-hop data forwarding, and edge computing are necessary operations for a robust swarm network [81]. Figure 9 shows a swarm of 3, 7, and 10 agents that were created to optimize area coverage under a shared network load for data transfer. 

**Figure 10:**
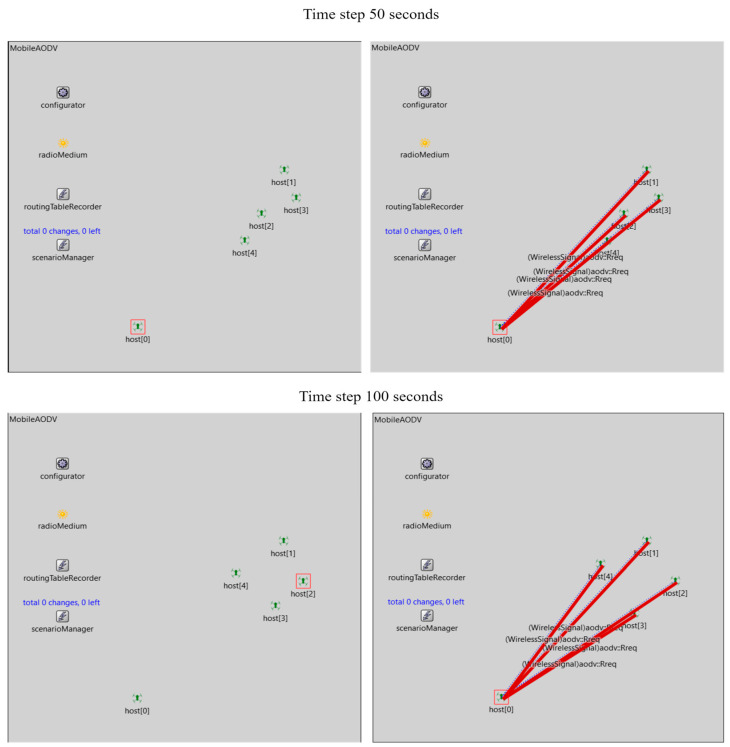
A mobile AODV routing protocol for a set of mobile and non-mobile agents using OMNET++.

Routing protocols form the basis of the network creation for UAV swarms for reliable node-to-node packet transmission, security, and energy efficiency. AODV (Ad Hoc On-Demand Distance Vector) is a self-starting routing protocol that has a degree of tolerance toward node failures [82,83,84]. A request–response cycle is initiated for hops. Although lacking in implicit security, it is used as the basis for future development of routing protocols [84]. Figure 10 shows interactions between a UAV agent acting as a leader and a set of mobile UAV swarm agents.

**Figure 11:**
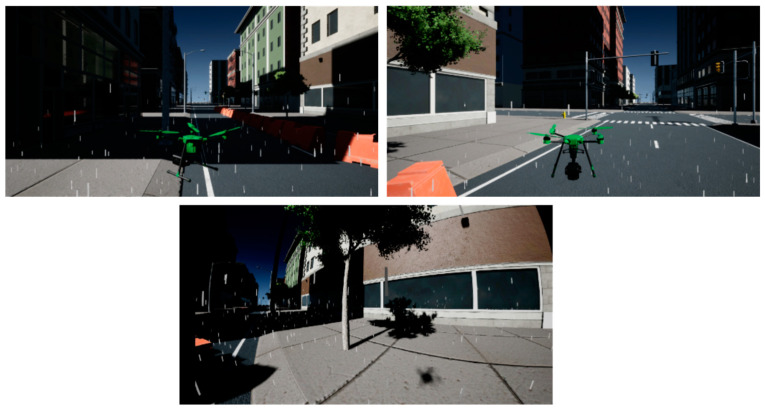
MATLAB simulation using the UAV Toolbox sub-figures on top show different angles of the simulated UAV and the bottom shows the feed from the fisheye image sensor.

For ES3, a single high-fidelity 9 DOF (Degree Of Freedom) drone equipped with a fisheye camera is simulated flying in a city block scenario. The UAV Toolbox works with MATLAB and Simulink platforms the implementation of mathematical constraints governing the aircraft model as well as simulation environment parameters. In this particular experiment, the weather was adjusted to fog and light rain. The Simulink model makes it possible to implement additional sensors such as LiDAR and ultrasonic sensors or tweak fuel level, GPS parameters, and aircraft framework physics. A randomly generated traffic light overlay was added as the targeted data for collection in the form of image data from the onboard sensor. Figure 11 shows the UAV agent simulation from different angles and a feed from the image sensor.

**Figure 12:**
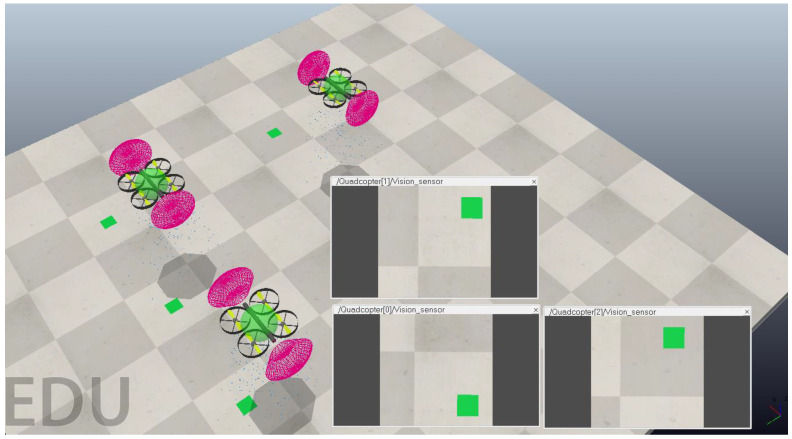
A 3-agent swarm simulated in MATLAB with a downward sensor for passive beacons and a limited-range lateral sensor to detect other agents.

ES4 experiments focused on swarm topology and inter-agent policies discussed above. Formation controllers can be further fine-tuned to adjust the allowable intrusion into an agent’s sphere of minimum distance. Low tolerance controllers record even the least amount of intrusion by one agent into another agent. These incidents are then noted by swarm control and corrective action for agent movement is taken. There is a fine tradeoff between the set tolerance and the computation power required. Low tolerance controllers promise lower collision rates; however, even minimum intrusions are examined. This results in more computational power used to examine intrusions and take corrective action. Higher tolerance controllers may not realize all intrusions except the most severe, freeing up computation power but opening up the risk for unpredictable collisions. Inaccuracies in measurement can be supplemented by additional methods. This can work for any sensor reading including GNSS (Global Navigation Satellite System) readings. A GNSS positioning system can be supplemented with localized passive beacons that transmit exact information, whereas drifts in agent movement can be compensated using formation control and additional sensors. Figure 12 shows this simulated approach.

**Figure 13:**
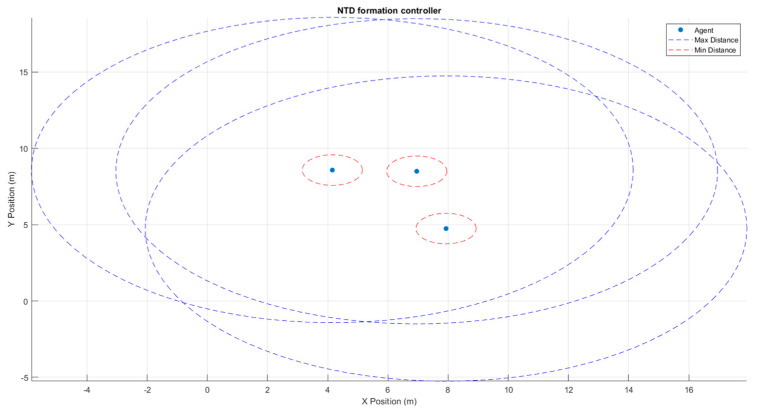
Three agents in an airspace with maximum and minimum inter-agent distances are set for formation control.

**Figure 14:**
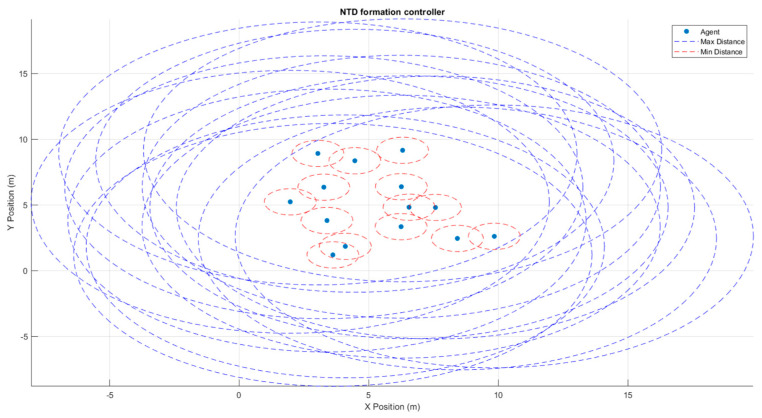
Fourteen agents flying the airspace with maximum and minimum inter-agent distances set for formation control.

Figure 13 and Figure 14 show three and fourteen agents in a swarm, respectively. A minimum and maximum distance between agents are set as means to complement other sensors and swarm formation methods. Based on the tolerance level set and the level of intrusion by one agent into the space of another, the controller alerts the possibility of an inter-agent collision such that suitable adjustments can be made to avoid it.

**Figure 15:**
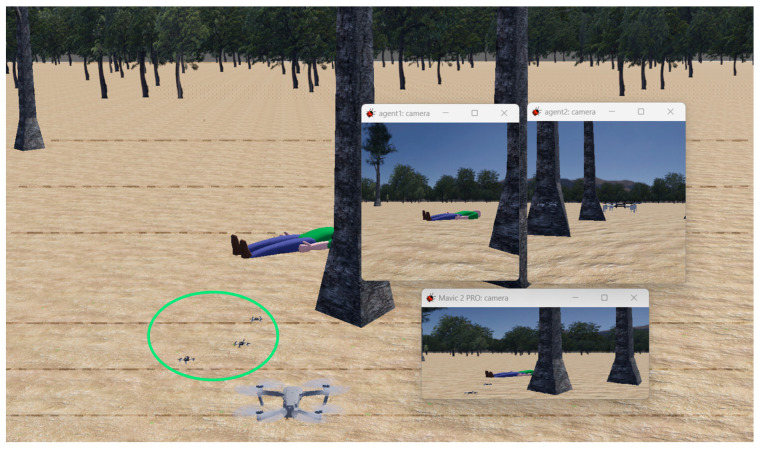
A distributed low-cost UAV swarm working towards targeted search and rescue simulated in a hyper-realistic environment in Webots.

An ES5 experiment has been designed in Webots for examining the application-specific functionality of a homogeneous swarm in a realistic environment. Here a swarm of low-level UAVs indicated by the green oval in Figure 15 work to locate a person in distress. An external agent controlled by an operator is also shown to provide an additional POV (Point Of View). Floating windows show an agent POV that has located the person in distress, a second POV from a different agent in the swarm, and the view of the manually controlled Mavic 2 camera following the swarm.

An additional pass-on module to MATLAB tracked agent positions to collect and visualize position data as a means of creating an efficient distance to network strength formation controller for maintaining inter-agent distances. The developed approach uses the concept of proportional changes in inter-agent network strengths as a result of changes in distance between them. APF (Artificial Potential Field) is used to create attraction and repulsion forces. The figure shows the Crazieflie [78] agents in a swarm converging on the person. Target search and detection applications such as this open a range of possibilities in disaster management and emergency response [5,85,86].

**Figure 16:**
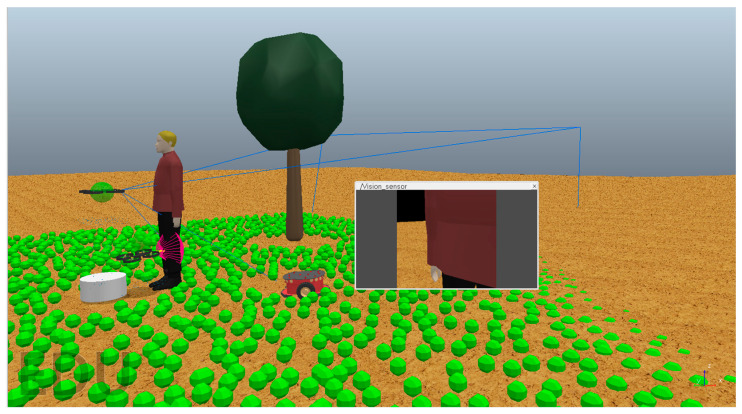
A heterogeneous swarm scenario with two UAV and one ground robot using CoppeliaSim.

The inclusion of agents with different capabilities in swarms is necessary to push the limitations of performance set by homogeneous swarms. A heterogeneous swarm can be recognized by various factors such as different operational spaces the swarm agents work in, the different nature of agents, or their hardware. An example of a swarm with all three properties was simulated in a CoppeliaSim simulation in Figure 16. An agent with a close-range sensor for obstacle detection works with an agent that has a long-range vision sensor; a ground-based wheeled robot is also a part of the swarm, and a tree is included as a sample for a static obstacle and a walking person is a dynamic obstacle. The aerial agents work in conjunction to detect obstacles and share information across the swarm. The ground-based robot is assigned different functions depending on the scenario such as acting as a platform for the safe landing of aerial agents or finding safe spots for the agents to land on the ground. The green spheres indicate possible zones for movement as mapped by the ground robot. Efficient information exchange pathways and the demonstration of heterogeneous agents with extended parameters are one of the targets for ES5. Simulation quality is enhanced with features such as obstacle variety, realistic scenery, and a mixed-sensor array. To design and test varied systems such as this, it is necessary for the right choice of simulation platforms that can support it.

Experiments beginning from ES3 use simulation platforms where it might be possible to introduce variability in experiments scenarios using weather effects. Rain, wind, and snow exert significant influence on UAV operations, underscoring the need for comprehensive testing. Rain can compromise UAV sensors and electronics, potentially disrupting communication and navigation systems. Wind poses challenges in maintaining stable flight paths and can increase energy consumption. Snow accumulation can alter UAV weight and aerodynamics, affecting maneuverability. The sun’s azimuth angles collectively shape the complex interplay of factors affecting UAV operations. The sun’s azimuth angle determines the direction of sunlight and casts shadows, impacting visual perception and sensor data accuracy. UAVs’ ability to interpret their environment and execute tasks can be compromised by glare, changing illumination, and altered shadow patterns. Properly accounting for the sun’s angle is crucial for accurate navigation, object recognition, and obstacle avoidance. All of these factors collectively impede control precision and reduce flight endurance, necessitating robust algorithms and hardware designs to ensure reliable UAV performance in adverse weather conditions. Integrating these environmental variables into UAV operation testing provides a comprehensive understanding of their combined impact, enabling the development of more robust and adaptable UAV systems capable of functioning effectively under a wide range of real-world conditions.

**Figure 17:**
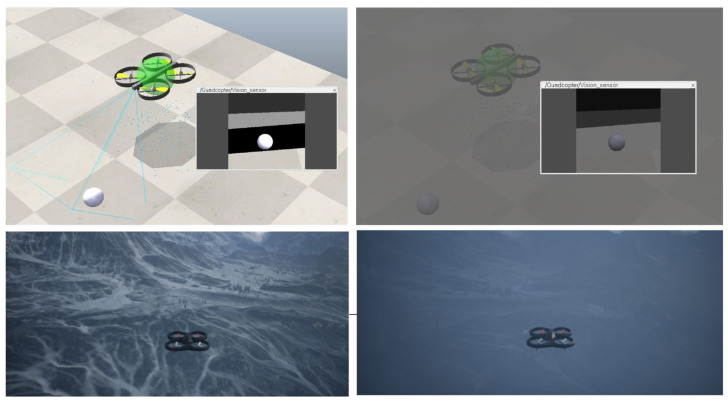
Top two figures show the effect of sun azimuth angle and fog affecting vision sensor capabilities. The bottom two figures show a UAV flying in clear weather (**left**) and in fog and snow weather (**right**) using Microsoft AirSim.

Figure 17 shows an example of how the presence of fog and changes in the sun’s angles may influence object detection by vision sensors. Adverse weather conditions may require the implementation of add-on methods to ensure that the output of sensors remain coherent [87]. The performance of other sensors such as LiDAR may also be affected. Table 5 summarizes some simulation platforms and the various weather effects that they are capable of supporting. Weather conditions such as wind, rain, and fog, can significantly impact the performance of UAVs, challenging their navigation, communication, and coordination capabilities. By incorporating weather effects, researchers can more accurately assess the resilience of swarm algorithms and control strategies under diverse environmental conditions. This approach not only provides a more comprehensive evaluation of UAV swarm behavior but also aids in identifying potential vulnerabilities and optimizing strategies for real-world deployment. Ultimately, the integration of weather variability lends a vital layer of complexity to UAV swarm experiments, promoting the development of more adaptable and robust swarm technologies.

The operating weather in UAV swarm experiments is an important factor as it enhances the realism and robustness of simulated scenarios. However, modifying graphical environment parameters may require additional development during the development phase of a platform as well as more computational resources while executing scenarios. The capability of a simulator to support different weather conditions can also be a crucial parameter that can assist in deciding which simulation platform to choose to perform experiment validation.

## 6. Future Work

**Figure 18:**
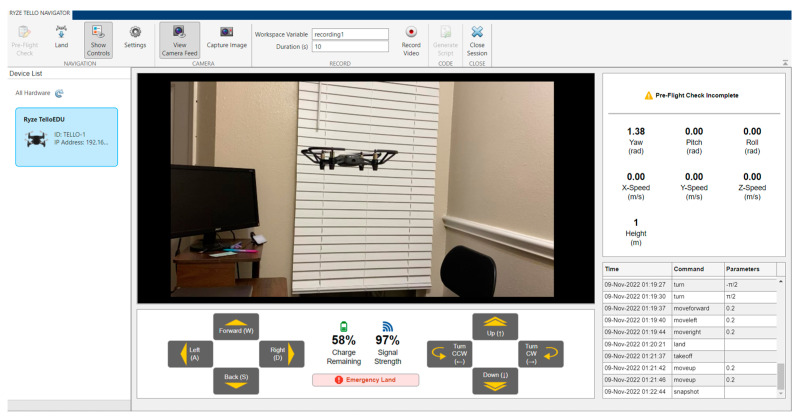
Ryze Tello package for Tello Edu drones.

A future research direction is to bridge the gap between simulation platforms and real-world UAV systems by establishing interfaces that enable bidirectional communication and synchronization. This would allow for the testing and validation of simulation results in controlled environments using fly nets and motion capture cameras. By integrating motion capture systems that accurately track and trace the movement of UAV agents, the collected data can be fed back into the simulation platform to validate and refine the simulated behaviors and algorithms. Modern simulation platforms or their associated packages allow for the transfer and deployment of code on supported hardware. There has been an increase in the number of platforms that hardware manufacturers support out of the box. The Ryze Tello package screen for MATLAB shown in Figure 18 supports Tello EDU drones. Control data can be exported for use and analysis. Multiple Tello drones in a swarm can also be programmed [88]. The easy interface of Tello drones with MATLAB and code functionality with Python, along with their low cost and swarming capability, were some of the reasons that they were chosen as the platform for conducting all hardware in-loop experiments by the authors. The framework in this article is not hardware specific and could be implemented using other platforms. The technology landscape is constantly evolving, and newer hardware such as the Robolink Codrone Edu platform introduced in a 2022 drone version offers similar capabilities to the Tello [89].

**Figure 19:**
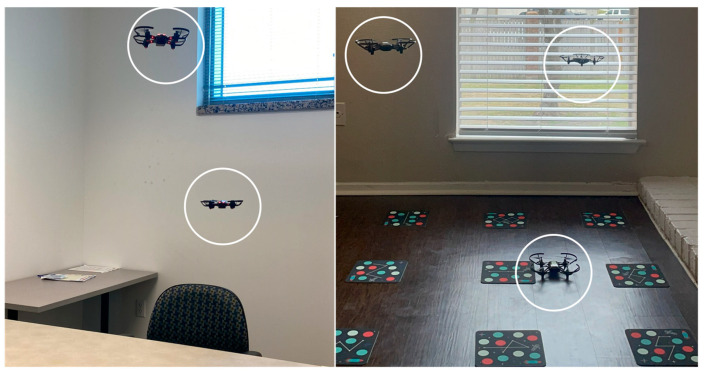
Two Robolink CoDrones flying in formation, (**left side**), and a swarm of three DJI Tello EDU drones including two active and one reserve agent (**right side**).

This closed-loop approach enables a continuous feedback loop between simulation and real-world experiments, enhancing the fidelity and reliability of the simulation results. Furthermore, the controlled environment provided by fly nets ensures the safety of the experiments while enabling the examination of intricate swarm behaviors and complex interaction dynamics. Such an approach would not only enhance the accuracy and realism of simulation results but also facilitate the transferability of findings from simulation to real-world applications. The ability to update simulation platforms also invites the possibility of allowing them to work with older hardware that supported the use of simulation tools but lacked certain features. A popular example is the now discontinued Robolink Codrone Pro platform which does not have swarm functionality, as seen in Figure 19–left. This platform was replaced by the Robolink Codrone Edu UAV. Swarming on the old version is still possible by creating custom scripts that pass position data to intermediaries. Every agent sends information to a node that updates other agents. Using this method, a decentralized proof-of-concept was created for the pro drone platform to enable agent swarming. Manufacturers now are more aware of the value of creating easily implementable bridges between simulation platforms and hardware. The DJI Tello EDU platforms now support swarm mode using MATLAB packages, as seen in Figure 19–right.

It is vital that the UAV designs themselves and the swarm experiments designed for them evolve to stay in line with new regulations enforced on the airspace that they fly in. The recently enforced rule by the FAA [90] mandates the presence of a RID module either as a modification to current hardware or by extension. The purpose of this module is to broadcast vital self-identifying information of the UAV and its controlling entity as a means to deter unauthorized activity, as well as to reduce load on conventional aircraft tracking measures. Simulation tools such as CoppeliaSim allow the creation of additional objects that can be fused to a prebuilt UAV and custom behavior can be defined for them. In this case, it would be the RID module periodically broadcasting information about the UAV such as position, task ID, and operator information. While this new rule has multiple concerns such as privacy concerns and extra costs, the data that it broadcasts can also be imbibed into existing swarm policies for resource tracking and management [91].

Future work should focus on developing standardized protocols and methodologies for integrating simulation platforms with motion capture systems and fly nets, enabling comprehensive and systematic validation of UAV swarm behaviors in controlled environments. Other than the experiment design itself, advances in the operation of UAV agents themselves should be explored. ANNs have been used to mitigate challenges with MBC design of flight controllers [92]. Similar approaches can be used to tweak experiment parameters that would enable observing a wider range of agent responses to introduced disruptions. This approach would significantly contribute to the advancement and practical applicability of UAV swarm research, fostering the development of robust and reliable autonomous systems for various domains, including surveillance, disaster response, and environmental monitoring.

## 7. Conclusions

The goal of the descriptive analysis presented here is to serve as a reference to other researchers who are currently working on similar swarm development experiments and are looking for various tools that might assist them in doing so. The contributions of this research lie in its thorough examination of simulation platforms and the proposed experimental design process. By providing a comprehensive guide for designing swarm experiments, the paper enables researchers to validate the robustness and efficiency of UAV swarm systems before field implementation. Also provided is a concise review of existing simulation platforms, assessing their suitability for swarm experimentation needs. This evaluation was used during the process of platform selection for designing swarm experiments. In future work, researchers can explore further advancements in simulation platforms, refine experimental design processes, and investigate novel swarm behaviors and coordination strategies. With continued research and evidence-based evaluation, UAV swarm systems can be further optimized, enabling their widespread implementation, and contributing to the advancement of autonomous aerial operations.

## Figures and Tables

**Table 1:** Recent works focusing on robotic simulators arranged by their published year.

Reference	Published Year	Description
[18]	2018	Comparing the performance of three popular robot simulators.
[21]	2019	Survey on UAV simulators, their features, and architecture.
[16]	2021	Survey on robotic simulators, platforms, and frameworks, with a distinction between the three terms.
[20]	2021	An overview of robotic simulators suitable for use in education.
[19]	2022	Review on swarm-focused simulators, real-life hardware, and applications.
This study	2023	An updated and comprehensive examination of simulation platforms capable of handling UAVs with an accompanying experimental design process for swarm-based research objectives.

**Table 2:** Simulation tools examined.

Name	OS Support ^1^	UAV Specific?	Possibility of Cross-Platform Connectivity ^2^	Notable Publication by Platform Creators	Remarks
Gazebo	W *, M *, L	No	Yes	—	—
Webots	W, M, L	No	Yes	—	Independent simulation platform with support for various robot platforms.
CoppeliaSim	W, M, L	No	Yes	[23]	Formerly known as VREP.
UAV toolbox (MATLAB)	W, M, L *	No	Yes	—	Although MATLAB is not UAV specific, the UAV toolbox is designed for UAV development.
RflySim	W	No	Yes	[28]	Independent simulation platform inspired by PX4 and MATLAB simulation platform.
ARGoS	M *, L	No	Yes^+^	[29]	Multiphysics robot simulator.
OMNET++	W, M, L	No	Yes	—	Discrete event simulation platform focused on networking and communication.
AVENS	W	Yes	Yes	[30]	Works with the OMNET++ network simulator and X-Plane flight simulator.
MORSE	W *, M *, L *	No	Yes	[31]	—

^1^ A * near the OS label indicates that direct binaries of the tool may not be available; however, alternatives such as docker deployments or community supported guides do exist. ^2^ A + near the yes indicates that the software has indirect passing of model data for cross-platform connectivity or the possibility of self-developed plug-ins.

**Table 3:** Recent work on UAV swarm development and the simulation platform they used is categorized by published year (Range 2021 to 2023).

Reference	Year Published	Study Description	Platform Used ^1^
[32]	2021	Formation control of heterogeneous UAV and USV swarms.	CoppeliaSim
[33]	2021	Architecture of UAV swarm to find a load and transport it to its destination cooperatively.	CoppeliaSim
[34]	2021	Adaptive formation control for UAV swarms with multiple leaders and switching topologies.	Gazebo
[35]	2021	Control of UAV agents in a swarm using vision-based approaches.	Gazebo
[36]	2021	Development of control layers to enable the autonomous and cooperative navigation of a swarm of UAVs.	Gazebo
[37]	2021	Optimized area coverage by autonomous multi-UAV.	Gazebo + MATLAB
[38]	2021	Approach to address coverage and flocking problems in multi-UAV.	Gazebo
[39]	2021	Bioinspired neural network for cooperative planning of multi-UAV.	Gazebo
[40]	2021	Safe allocation of UAV swarm mission resources based on random labels.	OMNET++
[41]	2022	Coverage and path planning algorithm for swarms to detect points of interest and collect information from them.	OMNET++
[42]	2022	A distributed UAV swarm formation system optimized by a hybrid evolutionary algorithm.	ARGoS
[43]	2022	Approach to optimized UAV swarm for improved intruder detection.	ArGoS
[44]	2022	A framework for simulating cooperative UAV swarms performing joint missions.	OMNET++
[45]	2022	Task decomposition and task correlation of UAV swarm	OMNET++
[46]	2022	Multiple UAVs collaborating for improved field phenotyping.	Gazebo
[47]	2022	Resource balancing for MAV (Mobile Aerial Vehicle).	Gazebo
[48]	2022	Development of a PSO-based threat avoidance and reconnaissance FANET.	Gazebo
[49]	2022	Entrap multiple targets using a robot swarm.	MATLAB + CoppeliaSim
[50]	2023	A decentralized method for multiple UAVs to explore separate areas.	Gazebo
[51]	2023	A novel IDS (Intrusion Detection System) that identifies deviation in normal UAV behaviors as means of indicating external threats.	Gazebo
[52]	2023	A multiple tracking methodology for aircraft at low altitudes.	MATLAB
[53]	2023	Collision avoidance strategy for D2D (Device to Device) communications in UAV networks.	MATLAB
[54]	2023	Evaluation of routing protocols in UAV ad hoc networks in SAR scenarios	MATLAB
[1]	2023	Autonomous cooperative mission planning for multiple UAVs conducting surveillance missions.	MATLAB
[55]	2023	Trajectory planning and formation maintenance in swarm using MPC and standoff algorithm.	MATLAB
[56]	2023	An improved PSO for optimized base station placement for UAVs.	CoppeliaSim

^1^ With regards to the use of MATLAB for experimental design, all cited references may not necessarily use the UAV toolbox, Simulink, or other add-ons, choosing to rely only on the main program. However, they are included in the table since the standalone MATLAB platform may be sufficient for their validation requirements.

**Table 4:** Experiment series with descriptions, target observations, and simulation platforms used.

Experiment Series (ES)	Description	Target Mechanisms Developed and Observed	Simulation Tools Used
1	Basic swarm phenomenon observation	Flocking maneuvers, 2D path planning.	MATLAB UAV Scenario Designer
2	Studying FANET topology and performance.	Examine inter-agent communication process, equipment range, and routing protocols.	OMNET++ and associated plugins.
3	Photorealistic Single UAV design and observation.	Examine the addition of various cameras, sensors on UAV, and data collection using simulated environments.	Webots, MATLAB UAV toolbox, & Simulink
4	Examining basic movement operations of UAV swarm.	Establishing and defining inter-swarm policies, agent deployment, and defining swarm topology	CoppeliaSim
5	Implementing ground terrain features as well as realistic obstacles with varied agents.	Defining buildings, trees, and realistic heterogeneous agents	CoppeliaSim, Webots

**Table 5:** A comparison of simulation platforms and the various weather effects they support.

Simulation Platform	Weather Effect	Additional Notes
CoppeliaSim	Limited to scripted animations	No built-in support for weather effects
Gazebo	Supports weather effects through plugins	Rain, fog, snow, and more can be simulated using community plugins
Webots	Simulates environmental conditions	Offers tools to adjust parameters like lightening, wind, and physics
UAV toolbox	Sun angle, time of day fog, and rain are possible by default	A slight variability in the mentioned weather factors is possible
Microsoft AirSim	A larger number of weather variations are possible.	In-built functions include controlling wind direction, rain, snow, dust, and fog.

## Data Availability

Data sharing is not applicable.

## Abbreviations

ANNs	Artificial Neural Networks
APF	Artificial Potential Field
AODV	Ad Hoc On demand Distance Vector
D2D	Device to Device
DOF	Degree of Freedom
ES	Experiment Series
FANET	Flying Ad Hoc Networks
GNSS	Global Navigation Satellite System
IDS	Intrusion Detection System
MAV	Micro Air Vehicle
MPC	Model Predictive Control
MANET	Mobile Ad Hoc Network
PSO	Particle Swarm Optimization
POV	Point Of View
ROI	Region of Interest
RID	Remote Identification
SAR	Search and Rescue
UAV	Unmanned Aerial Vehicle
UGV	Unmanned Ground Vehicle
USV	Unmanned Surface Vehicle
UAS	Unmanned Aircraft System
UWSV	Unmanned Water Surface Vehicle
